# Causal Modelling and Brain Connectivity in Functional Magnetic Resonance Imaging

**DOI:** 10.1371/journal.pbio.1000033

**Published:** 2009-02-17

**Authors:** Karl Friston

## Abstract

Recent advances in data analysis and modeling allow the use of fMRI data to ask not just which brain regions are involved in various cognitive and perceptual tasks, but also how they communicate with each other. Karl Friston examines two different state-of-the-art approaches to modeling brain connectivity using neuroimaging.

Neuroimaging studies that investigate the involvement of brain regions in various cognitive and perceptual tasks have become increasingly prevalent. Functional magnetic resonance imaging (fMRI) studies are especially popular, due to their non-invasive nature and high spatial resolution. With recent advances in data analysis and modelling, it is now possible to use fMRI data to ask not only which brain regions are involved in these tasks, but also how they communicate with one another; for example, one can ask, “Is attentional modulation of visually evoked responses mediated by top-down (task-driven) or bottom-up (stimulus-driven) connections in the brain?”

There are two state-of-the-art approaches for understanding the communication among distributed brain systems using neuroimaging. They reflect two distinct approaches to understanding connectivity. One approach—dynamic causal modelling (DCM)—tries to model how activity in one brain area is affected by activity in another (using models of effective connectivity), while the other—Granger causal modelling (GCM)—tests for the signature of these influences by looking for correlations in the activity of two or more regions (using models of functional connectivity). Previously, the relative accuracies of these methods, in disclosing patterns of communication among brain regions, were unknown. In a recent issue of *PLoS Biology*, Olivier David et al. compared them directly and provided evidence that may have a profound influence on their application [1]. Here, we consider the motivation behind the two techniques, their underlying assumptions, and the implications of David et al. [1] for their continued use.

## How Is the Brain Organised?

Most human brain mapping studies appeal to one of two principles of functional brain organisation: functional segregation and integration. Functional segregation posits a regionally specific selectivity for neuronal computations; for example, certain brain areas (e.g., V5 or MT) are specifically involved in processing visual motion. Functional integration, on the other hand, speaks to distributed interactions among functionally segregated regions. Studies of functional integration seek to understand how regional responses are mediated by connections between brain areas and how these connections change with experimental manipulations or disease. Functional integration is usually analysed in terms of functional or effective connectivity. fMRI provides measures of changes in blood supply to specific brain regions, in response to experimental manipulations (e.g., watching moving dots, relative to viewing stationary dots). Images of these haemodynamic responses are typically acquired every few seconds, producing a time-series of fMRI data at each point in the brain. Functional connectivity is defined as a statistical dependency between these regional responses over time (e.g., correlations in fMRI time-series or coherence in electromagnetic signals). Analyses of functional connectivity are concerned with the spatial deployment of these dependencies; in other words, which areas correlate with which other areas. On the other hand, effective connectivity is concerned with the directed influence one brain region exerts on another. This approach, unlike functional connectivity, tries to understand how one brain region affects another. To measure effective connectivity, one has to have a model of how this influence is mediated. Analyses of effective connectivity then try to quantify coupling in terms of the parameters of the connectivity model. In what follows, we will consider DCM and GCM in light of the above distinction between functional and effective connectivity.

## Causality and Coupling

In 2003, two techniques were introduced that addressed temporal dependencies and directed influences among distributed brain responses. These were DCM [2] and GCM [3,4]; both appeal to causality and rest on time-series models of fMRI data. However, beyond this, they differ radically in their ambitions and domains of application. We will look at these differences from the point of view of their underlying models, the inferences they afford, their implicit notion of causality, and their history.

## How Can One Model Brain Connectivity?

**Figure 1:**
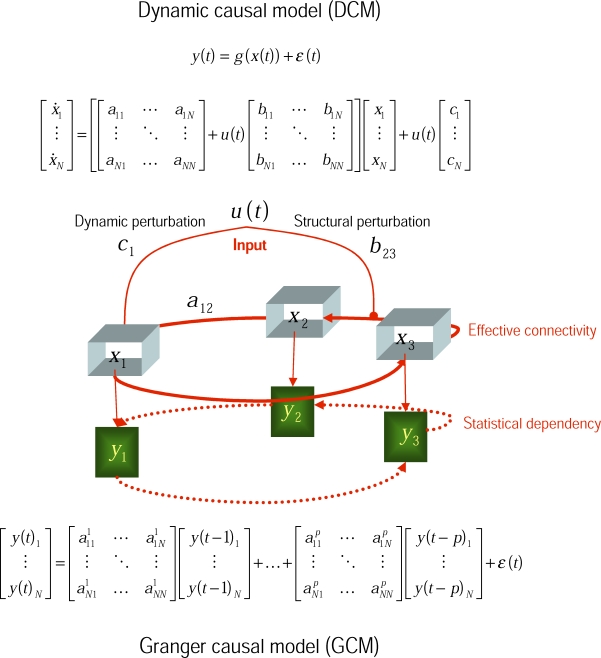
Models of Effective Connectivity This schematic shows the underlying equations on which dynamic (DCM) and Granger (GCM) causal models are based. In DCM for fMRI, bilinear differential equations describe the changes in neuronal activity *x*(*t*)*_i_* in terms of linearly separable components that reflect the influence of other regional state variables. Known deterministic inputs *u*(*t*) elicit a change in neuronal states directly though *c_i_* or increase the coupling parameters *a_ij_* in proportion to the bilinear coupling parameters *b_ij_*. The neuronal states enter a region-specific haemodynamic model to produce the outputs *y*(*t*)*_i_*. GCM tries to model the ensuing dependencies among the outputs with a time-lagged linear regression of the current response on previous responses (up to an order denoted by *p*). In both models, the data contain observation noise *ε*(*t*) that is added to regional observations. The DCM is effectively a state-space model formulated in continuous time; whereas the GCM is a vector autoregression model in discrete time. See Figure 2 for a fuller explanation of the haemodynamic part of the model.

**Figure 2:**
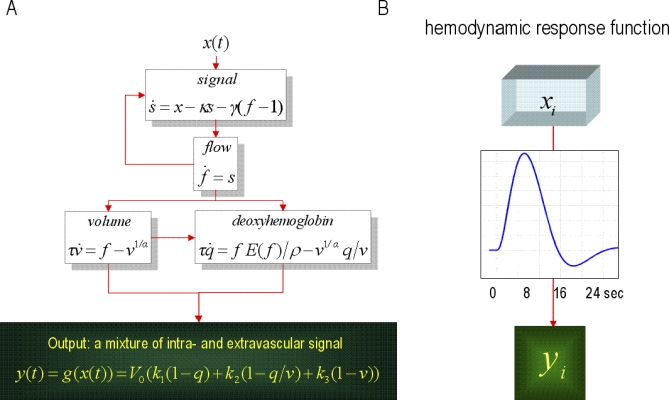
Modelling Haemodynamics (A) This schematic shows the architecture of a haemodynamic model for a single region. Neuronal activity induces a vasodilatory and activity-dependent signal *s* that increases blood flow *f*. Flow causes changes in volume and deoxyhemoglobin (*v* and *q*). These two haemodynamic states enter an output non-linearity to give the observed fMRI signal *y*. (B) This transformation from neuronal states *x*(*t*)*_i_* to haemodynamic response *y*(*t*)*_i_* is encoded graphically by the boxes in the previous figure and corresponds to a convolution. The implicit convolution kernel or haemodynamic response function is shown in the insert for typical values of the haemodynamic model's parameters.

The fundamental difference between DCM and GCM is that DCM employs an explicit forward or generative model of how observed data were caused. These models invoke hidden neuronal and biophysical states that generate data. In contrast, GCM rests upon a phenomenological model of temporal dependencies among the data themselves [5], without reference to how those dependencies were caused (see Figure 1). In this sense DCM is a model of effective connectivity, whereas GCM is used to infer functional connectivity. This distinction becomes crucial for fMRI, because fMRI signals are haemodynamic convolutions of underlying neuronal signals. In other words, the fMRI signals are the products of a complicated chain of physiological events that are initiated by changes in neuronal activity. This means that the observed fMRI response to a neuronal activation can be delayed and dispersed by several seconds. The convolution or impulse response function, mapping from underlying neuronal activity to observed fMRI responses, is called a haemodynamic response function and typically peaks at about four seconds (see Figure 2). DCM assumes haemodynamic signals are caused by changes in local neuronal activity, mediated by experimental inputs (e.g., the presentation of a visual stimulus or the instruction to attend to motion) and the distributed neuronal interactions among brain regions that ensue. DCM is based on a model of this distributed processing and is parameterised by the strength of coupling among the neuronal regions. This neuronal model is then supplemented with a haemodynamic model that converts the neuronal activity into predicted haemodynamic signals. For fMRI, the neuronal models are usually fairly simple and are based upon low-order approximations to otherwise complicated equations describing the evolution of neuronal states (see Figure 1). In contrast, the haemodynamic model is rather complicated (see Figure 2). Both the neuronal and haemodynamic parts of the DCM are specified in terms of non-linear differential equations in continuous time (hence dynamic). The parameters of these equations encode the strength of connections and how they change with experimental factors. It is these parameters DCM tries to estimate.

Conversely, the model used by GCM is formulated in discrete time and usually rests upon the assumption that any statistical dependencies among brain regions can be approximated by a linear mapping over time-lags (although more sophisticated non-linear models can be used; e.g., [6]). GCM has no notion of experimental inputs or evoked responses and assumes the fMRI signals are stationary and are driven by random fluctuations; see [7–9]. The parameters of their underlying regression models encode the degree of statistical dependence between regions and are simple regression coefficients. In summary, the models employed by DCM are complicated and domain-specific, in relation to the simple and generic models used in GCM.

## Why Are Models Important for Testing Hypotheses?

So why go to the trouble of creating realistic models of brain processes? Basically, because it allows one to compare different models or hypotheses about distributed neuronal computations. In DCM one fits or inverts the models by optimising (the distribution of) their parameters (i.e., connection strengths and other biophysical quantities like rate constants) with respect to the model's evidence. Put simply, one finds the distribution of parameters that renders the data the most likely, under the DCM considered. This optimisation furnishes two things. It provides the most likely parameters for any given model, and the model evidence itself. This evidence is simply the probability of observing the data under a particular model. The model evidence is a very important quantity because it allows one to compare different models and adjudicate among them [10]. In other words, it allows one to explore model space and find the best model that explains the data in a parsimonious way. If one equates each model with a hypothesis about the neuronal architectures subtending observed data, the model evidence provides a quantitative and principled measure for evaluating beliefs about different hypotheses. Once the best model has been selected, one can then look at its parameters and make probabilistic statements; such as, “attention selectively increased the top-down connection between …” Similarly, in GCM one compares two models with and without a directed mapping between brain areas. If the model with the mapping has more evidence than the model without, one can conclude that the mapping or dependency exists. This inference is usually the end-point of the GCM, because the parameters (regression coefficients) per se have no biophysical meaning. In summary, DCM enables model comparison over a number of competing hypotheses or models and inference on the biophysical parameters of the model selected. In GCM there are only two models, with and without a particular functional connection, and the object is to infer that this dependency exists. In both cases, establishing evidence for one model, in relation to another, allows one to declare some causal relationship; but is the nature of this causality the same?

**Box 1. The Most Cited Applications of Causal Modelling with fMRI**

These titles were identified by searching for “dynamic causal model*” and “Granger causal*” with “fMRI”; only application papers with ten or more citations are listed. Source: Web of Science.

- Effective connectivity within the distributed cortical network for face perception. Cereb Cortex (2007) 17: 2400–2406.
- Interhemispheric integration of visual processing during task-driven lateralization. J Neurosci (2007) 27: 3512–3522.
- Cerebral pathways in processing of affective prosody: A dynamic causal modeling study. Neuroimage (2006) 30: 580–587.
- Analyzing and shaping human attentional networks. Neural Netw (2006) 19: 1422–1429.
- Synaptic plasticity and dysconnection in schizophrenia. Biol Psychiatry (2006) 59: 929–939.
- Shifts of effective connectivity within a language network during rhyming and spelling. J Neurosci (2005) 25: 5397–5403.
- Dissociating reading processes on the basis of neuronal interactions. J Cogn Neurosci (2005) 17: 1753–1765.
- Investigating the functional role of callosal connections with dynamic causal models. Ann N Y Acad Sci (2005) 1064: 16–36.
- Where bottom-up meets top-down: Neuronal interactions during perception and imagery. Cereb Cortex (2004) 14: 1256–1265.
- A dynamic causal modeling study on category effects: Bottom-up or top-down mediation? J Cogn Neurosci (2003) 15: 925–934.

## What Is Causality?

Causality in GCM is used in a very colloquial fashion and does not mean the data from one part of the brain “cause” data in another part. This is why it is referred to as Granger causality (or G-causality). Conversely, causality in DCM is used in a control theory sense and means that, under the model, activity in one brain area causes dynamics in another, and that these dynamics cause the observations. Causality in GCM is based on temporal precedence and assumes the data reflect states that cause each other. Conversely, causality in DCM is based on the differential equations of motion, where activity in one brain region changes activity in another. Is this important? Provided one understands and qualifies the use of Granger causality, then probably not. However, it is a mistake to think that Granger causality implies a directed causal influence. Data cannot cause data; data are caused by underlying brain states. This issue is particularly acute for fMRI, where regional variations in the haemodynamic response function render the data acausal. Put simply, this means that neuronal activity occurring first in one area and then in a second may be seen in the haemodynamic responses in the second area before the first. This violates temporal precedence assumptions and could lead one to conclude that the neuronal target “Granger-causes” responses in the source. Why is this potential flaw in the temporal assumptions of GCM not widely appreciated?

## Where Do DCM and GCM Come From?

**Figure 3:**
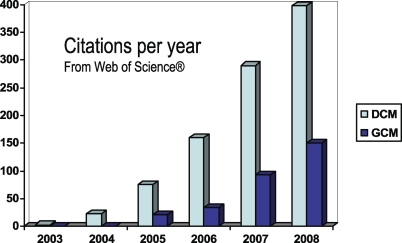
Comparative Citations Rates Citations rates for dynamic (DCM) and Granger causal modelling (GCM), since their introduction to fMRI in 2003. These citations were identified by searching for “dynamic causal model*” and “Granger causal*” with “fMRI”. Source: Web of Science.

Granger causality was developed in the social sciences and economics, where the complexities of real-time biophysical processes are not an issue. It should be noted that the general concept of Granger-Schweder causality [11] is based on martingale theory and is not tied to the specific linear autoregressive models in Figure 1. Martingales are stochastic or random processes that, in essence, have no memory (where their differentials are known as innovations [11]). Unfortunately, most random fluctuations in biological systems do have memory, because they are generated by dynamical systems. This confounds the application of GCM to biological time-series. The application of GCM to fMRI time-series coincided almost exactly with the inception of DCM. DCM is a newer approach to time-series data that was developed to overcome the limitations of conventional techniques (like structural equation modelling and GCM) and is based on systems theory. Both have enjoyed a fairly rapid growth in development and application (see Figure 3); for example, there are now dynamic causal models for electroencephalography (EEG), magnetoencephalography (MEG), tracer kinetics, and even local field potentials [12]. The sorts of questions addressed by causal modelling are exemplified by the selective bibliography in Box 1; and range from characterisations of hierarchies in language systems to lateralisation in visual processing.

**Box 2. Conceptual Motivations for DCM, in Relation To GCM**

- Granger causality among haemodynamic responses cannot be interpreted in terms of causal interactions among neuronal states. This is because variations in the haemodynamic response function over the brain violate temporal precedence assumptions.
- GCM tests models with and without connections. However, there are reciprocal polysynaptic connections between all brain areas, and models without connections are not appropriate null models.
- GCM tests whether a dependency is present or not. However, we already know connections are reciprocal; what we want to know is if the connection changes with experimental context.
- GCM (using vector autoregression models) rests on a linear mapping; whereas neuronal and haemodynamic processes are non-linear.
- GCM cannot accommodate experimental perturbations, because it models data, not the states that are perturbed.
- Granger causality cannot be used when stochastic terms (i.e., random fluctuations or martingales) are serially correlated, as in fMRI. This problem also confounds GCM of EEG and MEG data.

## An Empirical Validation of DCM and GCM

DCM was invented because of theoretical concerns about the application of GCM to connectivity in the brain (see Box 2). However, following the publication of David et al. [1], some of these conceptual issues are now empirical facts. This advance rests on an elegant study using multimodal techniques and a well-defined animal model of neuronal signal propagation and directed connections; namely a rat model of absence epilepsy with spontaneous spike and wave discharges. In brief, the authors were able to measure brain responses to sporadic epileptic events; both at their source (somatosensory cortex) and in connected brain regions. Critically, they measured both electrical and haemodynamic responses. This allowed them to infer the known connectivity using just the fMRI data (with DCM and GCM) and compare the estimates to the true connectivity based on electrophysiology. Furthermore, because they had a measure of the underlying neuronal (electrical) activity, they were able to assess regional variations in the haemodynamic response function that could confound GCM.

More specifically, David et al. recorded non-invasive EEG and fMRI signals during seizure activity and later recorded intracranial signals in the areas identified by the brain mapping. By recording both electrophysiological (neuronal) and fMRI (haemodynamic) responses, the authors were able to evaluate the haemodynamic response function empirically; in regions showing seizure-related responses. Critically, they found enormous differences between the haemodynamic response functions in different brain areas. The authors compared the results of DCM and GCM analyses of the fMRI data and showed that regional variation in the haemodynamic response function did indeed lead to different conclusions about the connectivity. They then went on to use the intracranial recordings to establish the face validity of the ensuing inferences. They did this by looking at the direction of neuronally mediated influences, in terms of delays and asymmetry in generalised synchronisation of these time-resolved measures. They were able to show that the driver of spike and wave discharges was correctly located in the somatosensory cortex when, and only when, haemodynamic effects were modelled appropriately by DCM.

This study highlights a key conceptual difference between DCM and GCM (see Table 1): namely, that DCM has an explicit model of hidden states causing observed data; whereas GCM tries to establish dependencies among the observations themselves. This is fine when the brain states that cause each other are observed directly (EEG), but not when the data are some post hoc consequence of these states (fMRI). David et al. illustrate this point by applying GCM to the fMRI data and then to the implicit neuronal activity. They show that the inferences are very different and that only GCM of implicit neuronal states gives sensible results. David et al. were able to do this because they had direct (EEG) measures that enabled then to undo (deconvolve) the haemodynamic effects and convert the fMRI data into a surrogate for neuronal states (using region-specific dynamic causal models and spike-wave inputs). In real-world fMRI applications, however, this would not be possible because one does not know the underlying neuronal activity. This is no problem for DCM because it assumes hidden neuronal states and models the haemodynamic convolution in each region explicitly.

**Table 1:**
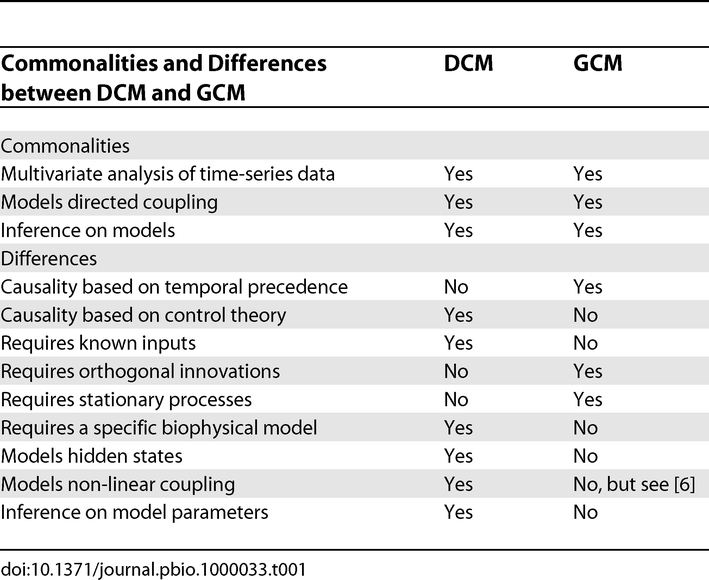
Comparing DCM and GCM

## Conclusion

The implications of David et al. [1] are far-reaching. They highlight the usefulness of well-defined animal models and multimodal recording. This work also provides one of the most potent empirical validations of DCM and, more generally, the attempt to model neuronal dynamics explicitly, when trying to explain brain imaging data. From the perspective of data analysis, it provides a clear pointer to the use of DCM over GCM and substantiates recent inferences about the confounding variability of haemodynamics over brain regions based on fMRI data alone [13]. At a more general level, it highlights the need for a clear understanding (and operational model) of how one thinks data are generated. Whether this is a good thing or not remains to be seen. DCM is a generic framework but is not based on a specific model; its application requires one to specify the way in which processes generating data are structured, in terms of differential equations. This contrasts with the simplicity of GCM, which can be applied directly to almost any time-series. One might think that obliging people to specify hypotheses about the way their data are caused is a good thing; but it does require a lot more investment and prior knowledge.

## Abbreviations

DCM: dynamic causal modelling
EEG: electroencephalography
fMRI: functional magnetic resonance imaging
GCM: Granger causal modelling
MEG: magnetoencephalography
